# Embryo Sign on Abdominal CT as the Footprint of Cecal Volvulus: Improved Practice Through Lessons Learned from a Case Report

**DOI:** 10.3390/life15121873

**Published:** 2025-12-08

**Authors:** Antonio Pierro, Alessandro Posa, Paolo Mirco, Dario Di Maio, Antonio Vallo, Marcello Lippi, Roberto Cristino, Pierluigi Barbieri, Pierpaolo Oriente, Antonio Totaro, Roberto Iezzi

**Affiliations:** 1 Department of Radiology, “S. Timoteo Hospital”, Regional Health Agency of Molise Region (ASRem), 86039 Termoli, Italy; antoniopierro.rad@gmail.com; 2 Department of Diagnostic Imaging, Radiation Oncology, and Hematology, A. Gemelli University Hospital Foundation, Istituto Nazionale Tumori Regina Elena (IRCCS), Largo A. Gemelli 8, 00168 Rome, Italy; dario.dimaio01@icatt.it (D.D.M.); marcello.lippi01@icatt.it (M.L.); pierluigi.barbieri@policlinicogemelli.it (P.B.); roberto.iezzi@policlinicogemelli.it (R.I.); 3 Department of Surgery, “S. Timoteo Hospital”, Regional Health Agency of Molise Region (ASRem), 86039 Termoli, Italy; paolo.mirco@asrem.molise.it (P.M.); antonio.vallo@asrem.molise.it (A.V.); roberto.cristino@asrem.molise.it (R.C.); 4 Health Directorate, “S. Timoteo Hospital” Regional Health, Agency of Molise Region (ASRem), 86039 Termoli, Italy; pierpaolo.oriente@asrem.molise.it; 5 Cardiology Unit, Responsible Research Hospital, 86100 Campobasso, Italy; antonio.totaro@unimol.it; 6 Department of Medicine and Health Sciences “V. Tiberio”, University of Molise, 86100 Campobasso, Italy; 7 Faculty of Medicine and Surgery, Università Cattolica del Sacro Cuore sede di Roma, Largo Francesco Vito 1, 00168 Rome, Italy

**Keywords:** abdominal CT, cecal volvulus, intestinal obstruction

## Abstract

Cecal volvulus is a rare cause of acute abdominal pain, resulting from torsion of the cecum and ascending colon due to abnormal mobility caused by inadequate peritoneal fixation. Clinical presentation is often vague and nonspecific, which can delay diagnosis and treatment. Computed tomography (CT) is the imaging modality of choice, as it not only confirms the presence and location of the volvulus but also identifies serious complications such as ischemia or perforation. Abdominal radiographs may be inconclusive, especially when the twisted bowel loop is fluid-filled, aligned antero-posteriorly, or obscured by adjacent gas-filled loops. We present the case of a 65-year-old woman who arrived at the emergency department with sudden-onset abdominal pain. Abdominal CT revealed classic signs of cecal volvulus, including the rarely reported ‘embryo sign,’ which proved crucial for swift diagnosis and intervention. Recognizing both common and less common CT features, such as the embryo sign, is paramount for rapid diagnosis and appropriate management in emergency situations. Familiarity with the full radiologic spectrum of this condition can significantly improve patient outcomes.

## 1. Introduction

Rokitansky first described cecal volvulus in 1837 [1]. This condition is rare and involves the twisting of the cecum. Cecal volvulus accounts for 11% of all intestinal volvulus, generally occurring in patients who are 30–60 years old. The age at presentation is influenced by the effects of dietary and cultural factors on intestinal mobility. An average age of 53 years has been reported in the western Countries, while 33 years in India and 52 years in Kenya [2].

Factors that can predispose someone to cecal volvulus include excessive cecal mobility, long or lax mesentery, constipation, hyperfixation of the ileum or colon, advanced age, a high-fiber diet, previous surgeries that cause adhesions, pregnancy [1,3,4,5,6,7,8,9], the presence of an abdominal mass, or scarring from calcified lymph nodes [10]. The embryological development of the intestine follows a complex, stepwise progression. In its final stages, the cecum undergoes a counterclockwise rotation, moving from the left side of the abdomen to its definitive location in the right lower quadrant. Concurrently, the mesentery of the right colon becomes anchored to retroperitoneal structures. In individuals with incomplete intestinal rotation, this fixation may be insufficient, predisposing them to cecal volvulus.

Concerning the various types of twists, approximately half of the patients experience a twist in the cecum, which rotates clockwise or counterclockwise around its long axis in the axial plane [10]. This twist is commonly observed in the right lower quadrant (type I); the other half of the patients exhibit the “loop type” of cecal volvulus, the variant we present, characterized by the simultaneous twisting and inversion of the cecum. In the latter type, the cecum appears to be situated in the left upper quadrant of the abdomen. The diagnosis may be confirmed by visualizing an appendix that is atypically placed and packed with gas (type II). A recognized variant of cecal volvulus is known as a ‘cecal bascule,’ characterized by anterior folding of the cecum in the absence of axial torsion. This condition typically presents as a distended bowel loop located in the central abdomen (type III) [11].

The onset can be sudden or preceded by recurrent and intermittent abdominal pain, leading to acute intestinal obstruction. Severe rotation can cause life-threatening complications such as vascular compromise, parietal necrosis, and perforation. The recurrent, intermittent presentation is also known as “mobile cecum syndrome”. This clinical pattern has been documented in approximately half of patients prior to the development of acute cecal volvulus. It is typically characterized by episodes of abdominal discomfort—either diffuse or localized to the right lower quadrant—accompanied by abdominal distension, with symptom relief following the passage of flatus. Patients presenting with acute cecal volvulus and bowel obstruction often exhibit clinical features that closely mimic those of uncomplicated small bowel obstruction. If left untreated, acute cecal volvulus can progress to intestinal strangulation and perforation, resulting in a fulminant clinical course. In such cases, patients commonly present with intense abdominal pain, signs of peritoneal irritation, dehydration, and hemodynamic instability [12].

Diagnosing cecal volvulus is challenging due to its nonspecific symptoms, and radiological diagnosis in an emergency setting is complex [1,3,4,5,6,7,8,13].

Among the well-documented signs of cecal volvulus detected by computed tomography (CT), the “embryo sign,” although present, is rarely explicitly mentioned [2,14,15,16,17,18]. Pathognomonic CT findings of cecal volvulus include abnormal position and distension of the cecum, the “coffee bean”, the “bird beak”, the “whirl” sign, a central appendix, and distal bowel collapse [10,19]. The term “coffee bean” sign typically describes an axial CT image showing a distended cecum filled with air and fluid, which can appear in various regions of the abdominal cavity, although it is more often associated with sigmoid volvulus. The “bird beak” appearance refers to the gradually narrowing afferent and efferent bowel loops that converge at the point of torsion. The “whirl” sign is used to characterize a CT finding where a soft tissue mass displays a swirling internal pattern made up of soft tissue and fat densities. In cases of acute cecal volvulus, this whirl pattern consists of a twisted, collapsed cecal loop, surrounded by low-density mesenteric fat and congested mesenteric vessels. Additionally, a gas-filled appendix may sometimes be observed, which has been associated with cecal distension due to volvulus [12].

Abdominal radiographs may not always provide a precise diagnosis, as up to 30% of patients may show no abnormalities on X-ray, which can contribute to delayed recognition of the condition, so computed tomography (CT) is the preferred imaging modality for identifying volvulus and its potentially fatal complications, such as ischemia and perforation [19,20].

Surgical intervention is the sole effective approach in treating cecal volvulus [19]. In case of complications like gangrene or perforation, surgical resection becomes imperative. Current surgical treatments involve options such as manual untwisting of the bowel, fixation of the cecum (caecopexy), creation of a cecostomy, or removal of the affected bowel segment (colectomy), which can be performed using either open surgery or minimally invasive laparoscopic techniques.

We describe a case of acute abdomen due to cecal volvulus where the “embryo” sign (or cecum in a fetal position) enabled a swift diagnosis and treatment. Our presentation referred to type 2 cecal volvulus, where the cecum and terminal ileum twist, positioning the cecum abnormally in the left upper quadrant [21].

This sign, through a heuristic vision of the CT findings from the early stages, helped to facilitate the interpretation and management of a complex clinical case in an emergency scenario.

## 2. Detailed Case Description

The patient, a 65-year-old woman, had been taken to the emergency department because she was experiencing intense, sudden abdominal pain accompanied by abdominal swelling and constipation lasting for approximately 9 h; she had no prior medical history of note.

Type II diabetes mellitus and long-standing arterial hypertension were revealed in the patient’s medical history.

Upon abdominal palpation, a resonant mass was detected in the left hypochondrium, accompanied by diffuse tenderness but without any sign of peritoneal irritation. The laboratory results showed elevated C-reactive protein level (59 IU/mL), average white blood cell count, and functional renal failure.

Pertinent laboratory tests reflected metabolic alkalosis, likely related to fluid sequestration in the dilated intestine, with a suboptimal hemodynamic status, suggested by hypovolemia and tachycardia.

Using an Optima 64 slice CT scanner (GE, Healthcare, Chicago, IL, USA) with a slice thickness of 1.25 mm, an unenhanced computed tomography of the abdomen was carried out. Contrast medium was not deemed necessary due to the patient’s reduced renal function and the results of the unenhanced CT.

**Figure 1:**
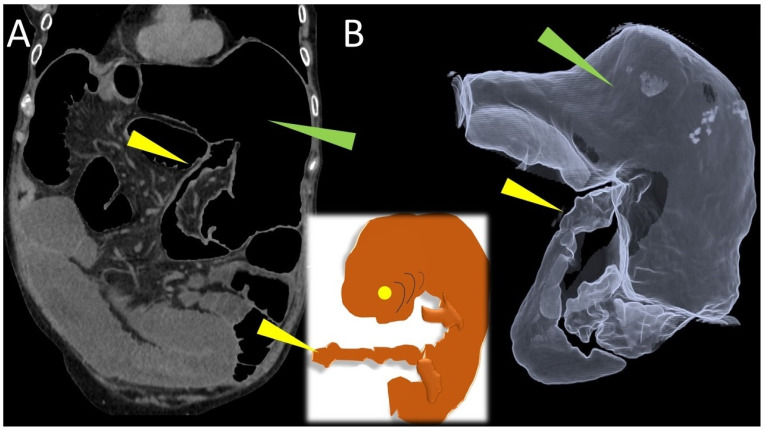
Coronal (**A**) CT scan shows the cecal volvulus, loop type; the caecum, greatly distended with air (green arrowhead), was inverted and filled the upper left region of the abdomen. Three-dimensional volume rendered (**B**) image of computed tomography, simulates a double-contrast barium study, demonstrated the cecal volvulus (green arrowhead), ileocecal valve and terminal ileum (yellow arrowhead).

A radial torsion of the ascending colon with terminal ileum resulted in a closed-loop pattern blockage of the caecum, as revealed by abdominal CT imaging. The cecum, markedly distended with air, appeared inverted and occupied the upper left quadrant of the abdomen, exhibiting a morphology reminiscent of a 7–8-week-old fetus, complete with a cord-like structure (Figure 1).

**Figure 2:**
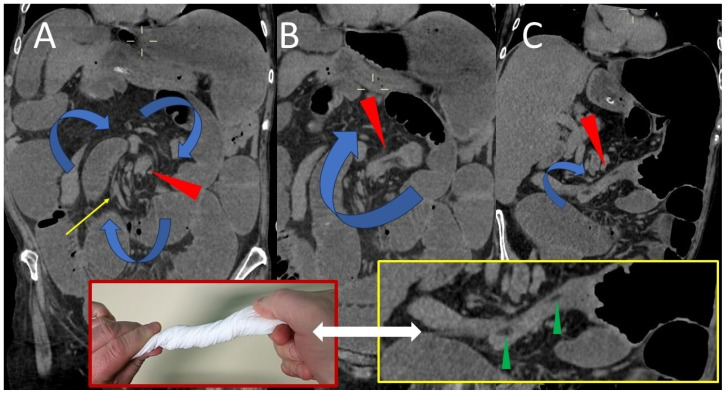
Coronal (**A**) CT image demonstrates a vortex composed of the cecum entangled on its longitudinal axis and a spiral of mesenteric vessels (yellow arrow) rotating consensually with the cecum: the “whirl” (red arrowhead). Seen along the longitudinal course, the entangled cecum (red arrowheads in (**B**,**C**)) contains small hypodense areas between its collapsed and intertwined walls, which represent mesenteric tissue incarcerated by the spiral braid of the cecal walls (green arrowheads in the yellow box).

**Figure 3:**
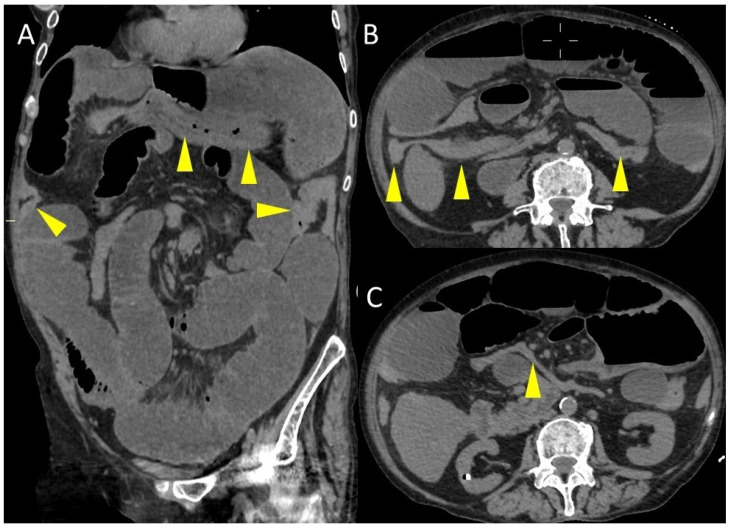
Coronal (**A**) and axial (**B**,**C**) CT images. The yellow arrowheads indicate the colon, which is collapsed throughout its course.

The CT scan also revealed the characteristic mesenteric whirl signs, a dilated cecum with an air/fluid level, marked overdistension with air-fluid levels of the small bowel and the collapsed appearance of the entire colon (Figure 2 and Figure 3). The cecum becomes tangled in the mesenteric vortex, just like a tissue woven paper along its longitudinal axis by opposing rotary movements of the hands.

The patient’s suboptimal hemodynamic status increased risk of complications. This led to the surgical option after imaging revealed cecal volvulus.

**Figure 4:**
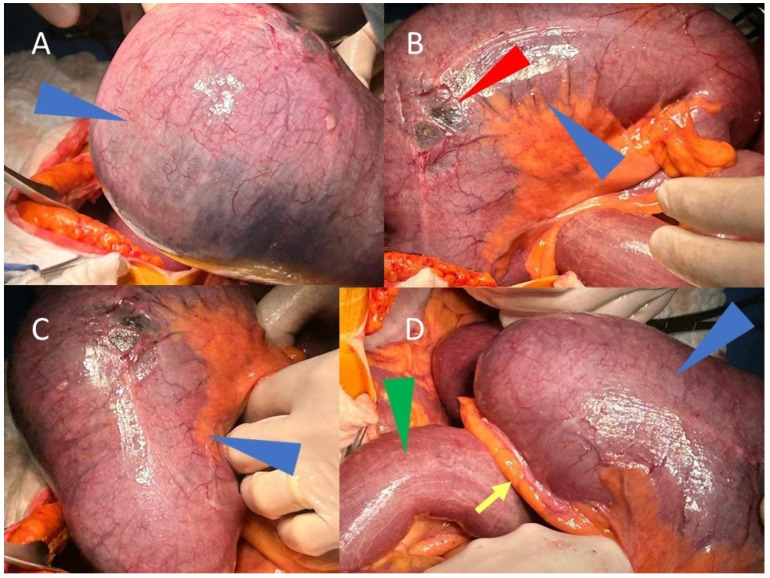
Three-dimensional volume rendered CT image, with parameters optimized for representation of aerial structures, show the cecal volvulus and terminal ileum with the morphological appearance of a 7–8-week fetus with its cord. Intraoperative images (**A**–**D**) show, in different angles, the volvulus of the cecum (blue arrowheads) and the terminal ileum (green arrowhead in (**D**)), both markedly overdistended. The vermiform appendix is visible (yellow arrow in (**D**)), while in B, the diastasis of the overdistended cecum is evident (red arrowhead).

The right iliac fossa was found to be empty during the emergency laparotomy exploration. Furthermore, as illustrated in Figure 4, a cecal volvulus affecting the ascending colon and terminal ileum was observed.

There were no signs of intestinal necrosis, but there were clear signs of diastatic rupture of the cecum resulting from mechanical obstruction with many serosal tears due to the distension.

The presence of serosal tears and the considerable risk of perforation made conservative or endoscopic detorsion hazardous, even though ischemia was not seen; therefore, a right hemicolectomy with a mechanical latero-lateral anastomosis was therefore considered the best course of action. No intraoperative or postoperative complications occurred.

The postoperative course required hospitalization in intensive care for four days due to comorbidities before being transferred to the surgery department until discharge from the hospital in good general clinical conditions.

## 3. Discussion

Cecal volvulus is a rare cause of intestinal obstruction with vague and nonspecific symptoms, and CT scans are the preferred imaging modality to enhance early detection [22]. Insufficient knowledge about this condition plays a role in delays in diagnosis and treatment.

A common cause of cecal volvulus is an aberrant fixation of the cecum, which causes the cecum torsion along one of its three axes [23]. The torsion of the ascending colon and cecal-ileum complex around its mesentery results in a closed-loop obstruction [24]. This condition accounts for 1–1.5% of adult intestinal obstruction cases, and a 31% mortality rate can result from a delay in diagnosis [24].

A higher risk has been linked to certain reported factors such as an history of abdominal surgery that may cause intestinal adhesions, pregnancy, excessive fiber intake through diet, chronic constipation, adynamic ileus or distal bowel obstruction, the presence of an abdominal mass or scarring from calcified lymph nodes, and the use of antipsychotic drugs with anticholinergic, serotonergic, and noradrenergic effect [10,25,26,27]. A recent review of cecal volvulus in children has confirmed the rarity of the condition in this population, even though a higher predisposition to cecal volvulus can be observed in children with mental disability (13/40 patients), probably due to abnormal peristalsis, tendency to aerophagia and constipation, and previous abdominal surgery [28,29,30]. In a study by Amra and colleagues, cecal volvulus was found to be associated with Hirschsprung’s disease in around 42% of cases [31]. Cecal volvulus has also been reported as a post-partum occurrence, probably due to associated colonic hypermobility and rapid change in uterus size [32,33].

Less than 20% of cases can be diagnosed through plain abdominal X-ray, while history, examination, and blood tests are nonspecific [25].

Patients suffering from acute cecal volvulus who do not receive adequate and prompt medical treatment may develop intestinal perforation and strangulation, which can present with sudden and severe symptoms [19]. These symptoms usually include severe abdominal pain with peritonitis and haemodynamic instability [12].

CT is the most comprehensive diagnostic modality to evaluate cecal volvulus and has the added benefit of detecting potential complications such as ischemia or intestinal perforation. Abnormal distension of the malpositioned cecum, gaseous distension of the centrally placed appendix, presence of the “whirl sign” and collapse of the distal bowel are considered pathognomonic CT signs [34]. Moreover, CT imaging can reveal features suggestive of intestinal ischemia or necrosis, including submucosal edema, reduced or absent enhancement of the bowel wall, the presence of pneumatosis intestinalis, or evidence of perforation such as free intraperitoneal air [35] and it also allows a major distinction between the three pathophysiological kinds of cecal volvulus [10].

The presented case involves a cecal volvulus in the left upper quadrant, causing acute intestinal obstruction. Axial torsion affecting the ascending colon, terminal ileum, and cecum is the defining feature of this condition. In this particular case, the distension and anomalous positioning of the cecum, the inversion of the position of the ileocecal valve and the terminal ileum, together with the presence of a consensual vortex sign, are key pathognomonic findings observable at examination of CT images.

The timely diagnosis and immediate surgical intervention are crucial in managing cecal volvulus. Failure to promptly diagnose the condition can result in intestinal necrosis or perforation, which can significantly worsen the prognosis, especially in elderly patients [19].

Conservative treatment is not recommended due to the elevated risk of ischemia, while surgical detorsion by itself presents a significant likelihood of recurrence [36].

Surgical intervention remains in most cases the treatment of choice, with the specific approach determined by factors such as the patient’s condition and findings during the perioperative period. The ongoing advancements in laparoscopic techniques have significantly reduced postoperative complications [37]. In particular, surgical techniques like cecopexy can be very useful in the management of cecal volvulus in children to prevent recurrence [28]. A recent multicentre study by Rasilainen and colleagues revealed that ischemic/necrotic bowel and co-morbidities can predict death in patients with caecal volvulus, therefore underlining the need for the individuation of early signs of volvulus at diagnostic imaging [38].

The majority of research suggests that it is crucial to initiate treatment within a timeframe of 24–72 h following diagnosis. This specific duration allows for proper hydration and necessary investigations. Despite timely and successful treatment of cecal volvulus, morbidity remains high due to prolonged ileus or respiratory failure [21].

Intestinal obstruction remains a cause of high mortality and morbidity. Identifying the cause of obstruction and the causal scenario remains a complex challenge for the radiologist, as identifying the cause of the obstruction can be a daunting task.

In this context, the use of radiological signs that can support and corroborate the diagnosis is crucial for effective and timely management of the emergency [39,40].

Specifically, the performance of some radiological signs in corroborating the diagnosis of cecal volvulus is well known. In contrast, other signs such as the embryo sign are rare but should not be considered stepchildren in the diagnostic effort [16,41,42].

The “embryo sign” represents an unusual imaging feature occasionally associated with cecal volvulus.

Unlike the well-established CT findings that are most often described in the literature, such as the whirl sign or the bird-beak appearance, this sign has received only limited attention and is rarely documented in clinical practice.

Its rarity, however, should not diminish its diagnostic value: when it is recognized, the sign can provide a meaningful contribution to the radiologist’s interpretation, particularly in acute emergency settings where rapid and accurate diagnosis is essential.

Published reports emphasize that the embryonic sign has been mentioned only in a relatively limited number of case reports and reviews, which explains its limited use among commonly used CT criteria; This type of cecal volvulus, which resembles the morphological appearance of a 7- to 8-week-old fetus with its umbilical cord, is clearly recognizable in the iconography of many clinical cases reported in the literature [15,32,43,44,45,46,47,48], although in many analyzed cases it is not explicitly mentioned as the embryo sign.

Highlighting these less familiar features underscores the broader principle that radiologists must remain alert to subtle or infrequent markers that may aid in diagnosis.

In our experience, this radiological sign has been very useful for a timely diagnosis. Its visibility, particularly in the scout view and coronal reconstructions, of a visceral sac over-distended with air, with a kidney-like morphology resembling an embryo, in an atypical location, allowed for a preliminary heuristic diagnosis corroborated by all the other findings present, such as the abnormally positioned terminal ileum, the completely collapsed colon, and the whirlpool sign.

We believe it prudent to underline that the identification of an overdistended intestinal loop with isolated embryo-like morphology cannot be considered a necessary and sufficient condition to unequivocally diagnose cecal volvulus unless all the additional signs that distinguish the biomechanics of the volvulus can be demonstrated.

Heuristically, this sign allowed us to realize the probable diagnosis from the early stages of the CT examination, facilitating its interpretation and allowing for rapid management.

Sharing our experience, we present an additional practical tool that may aid in the identification of cecal volvulus in real-world emergency imaging scenarios, also helpful in differential diagnosis from other causes of colonic obstruction (i.e., Ogilvie’s syndrome, cecal bascule) [49,50]. Finally, regarding the choice of the type of treatment adopted in our case, the surgical choice was considered after the suspicion of cecal volvulus on imaging, in relation to the patient’s hemodynamic status and her many comorbidities.

Although non-operative colonoscopic decompression is feasible, its low success rate makes it unwise [9,51].

Even in cases where necrosis is not present, surgical resection and anastomosis are advised as the first line of treatment to avoid recurrence with the least amount of side effects and mortality [9,51,52].

## 4. Conclusions

The embryo sign, characterized by the torsion of the cecum and terminal ileum with the cecum located ectopically and inversely oriented, is rare but could be a valuable CT finding that supports the diagnosis of cecal volvulus despite more widely recognized signs of cecal volvulus. Although rare, this sign should not be ignored in favor of all the other traditional and well-known CT signs of cecal volvulus, lest a valuable diagnostic opportunity be missed.

## Data Availability

No new data were created or analyzed in this study.

## Abbreviations

The following abbreviations are used in this manuscript:

CT	Computed Tomography
